# Supplementary material for: Spatio-temporal estimation of wind speed and wind power using extreme learning machines: predictions, uncertainty and technical potential
Source: Stoch Environ Res Risk Assess. 2022 Jul 12;36(8):2049–69. doi: 10.1007/s00477-022-02219-w (PMC9463360; doi:10.1007/s00477-022-02219-w)
Supplement: Supplementary file 1 — Supplementary file1 (PDF 35308 kb) [file 477_2022_2219_MOESM1_ESM.pdf]

Appendix to "Spatio-temporal estimation of wind speed and wind power using Extreme Learning Machines: predictions, uncertainty and technical potential"

Federico Amato[1*†], Fabian Guignard[2†], Alina Walch[3], Nahid Mohajeri[4], Jean-Louis Scartezzini[3] and Mikhail Kanevski[5]

[1*]Swiss Data Science Centre, École polytechnique fédérale de Lausanne (EPFL) and Eidgenössische Technische Hochschule Zurich (ETH), Switzerland.
[2]Institute of Mathematical Statistics and Actuarial Science, University of Bern, Switzerland.
[3]Solar Energy and Building Physics Laboratory, Ecole Polytechnique Fédérale de Lausanne, Switzerland.
[4]Institute of Environmental Design and Engineering, Bartlett School of Environment, Energy and Resources, University College London, UK.
[5]Institute of Earth Surface Dynamics, University of Lausanne, Switzerland.

*Corresponding author(s). E-mail(s): federico.amato@epfl.ch;
Contributing authors: fabian.guignard@stat.unibe.ch; alina.walch@epfl.ch; nahid.mohajeri.09@ucl.ac.uk; jean-louis.scartezzini@epfl.ch; mikhail.kanevski@unil.ch;
[†]These authors contributed equally to this work.

Appendix A

A.1 Exploratory Data Analysis

This section shows the main outputs of the exploratory data analysis performed on the wind speed data. Spatial plots and time series are used to highlight the presence of spatio-temporal structures and dependencies in the data. Temporal correlation are further explored for a set of stations located at different altitudes via autocorrelation functions (ACF). Distributional properties are explored using a kernel density estimation (KDE).

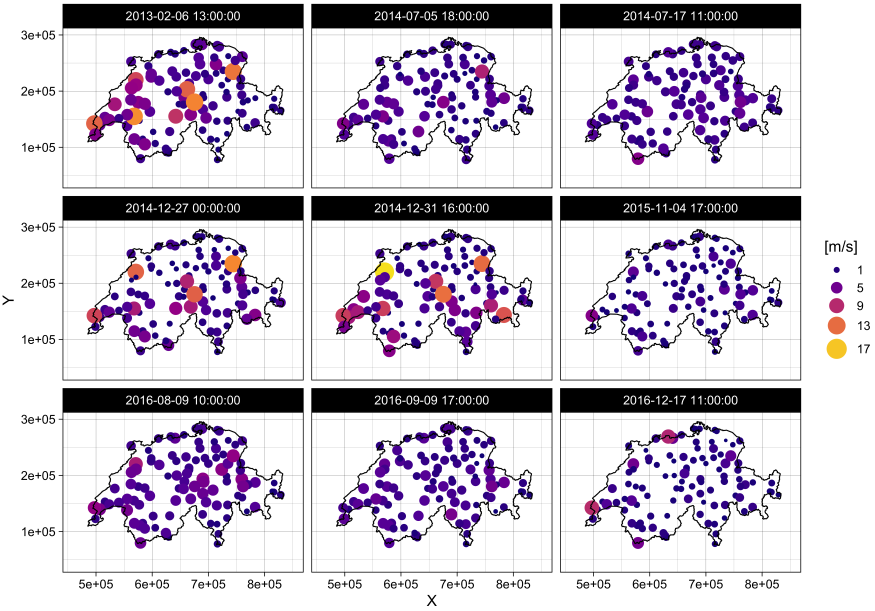

Fig. A1: **Spatial snapshots.** Spatial plots at different times for MSWind 13-16. The wind speed values are described by both, colour and dot size.

**Name**	**Location**	**Altitude [m]**
AND	Andeer	987
COM	Acquarossa Comprovasco	575
DAV	Davos	1594
DOL	La Dôle	1669
ENG	Engelberg	1035
EVI	Evionnaz	482
FAH	Fahy	596
INT	Interlaken	577
LUZ	Luzern	454
NABBER	Bern	535
NABDUE	Dübendorf	432
SCM	Schmerikon	408
STG	St. Gallen	775
TSG	Arosa (Tschuggen)	2040
ZER	Zermatt	1638

Table A1: **Some monitoring stations of MSWind 08-12.** Locations and altitudes for randomly chosen stations used in Figures A2, A3 and A4.

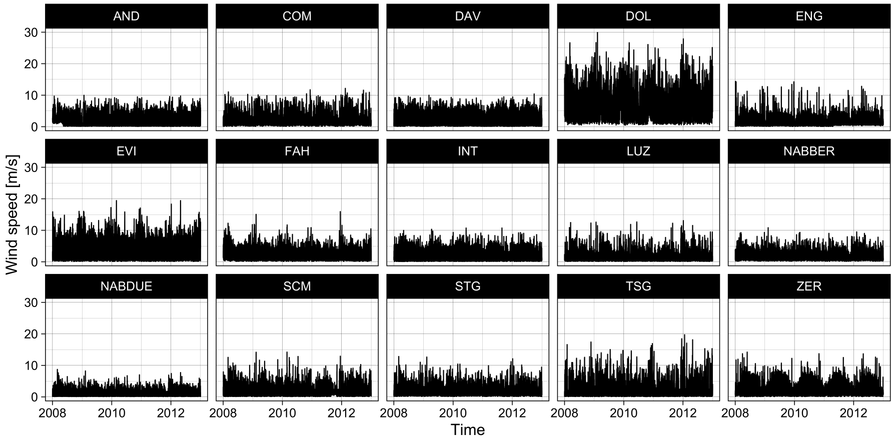

Fig. A2: **Time series plots.** Wind speed measurements of stations presented in Table A1.

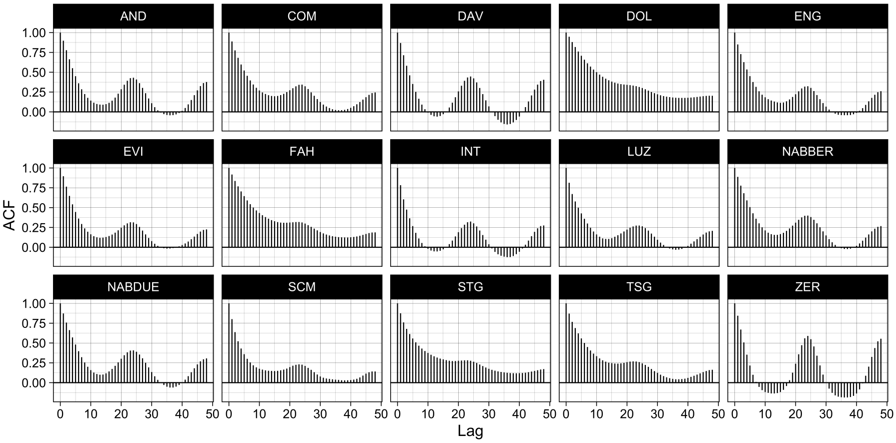

Fig. A3: **ACFs.** Wind speed ACF of each stations presented in Table A1.

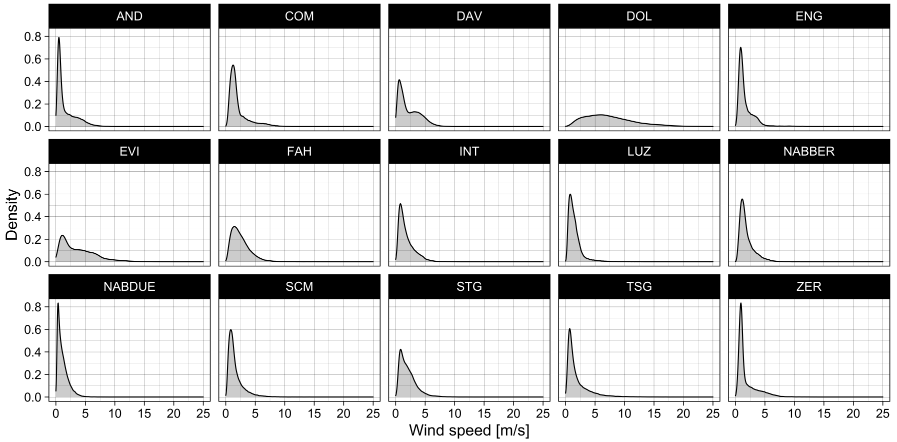

Fig. A4: **KDEs.** Each wind speed distribution of stations presented in Table A1 is estimated with KDE.

A.2 Input Features

This section provides details on the features included in the input space and the corresponding bandwidth parameters used to extract them from the digital elevation model. A correlation matrix showing relationships among pairs of features is also reported.

ID	Feature	Bandwidth
3	Difference of Gaussian (small scale)	$\sigma_1 = 0.25\ km, \sigma_2 = 0.05\ km$
4	Difference of Gaussian (medium scale)	$\sigma_1 = 1.75\ km, \sigma_2 = 2.25\ km$
5	Difference of Gaussian (large scale)	$\sigma_1 = 3.75\ km, \sigma_2 = 5.55\ km$
6	Slope (small scale)	$\sigma = 0.20\ km$
7	Slope (medium scale)	$\sigma = 1.75\ km$
8	Slope (large scale)	$\sigma = 3.75\ km$
9	Directional derivative N-S (small scale)	$\sigma = 0.25\ km$
10	Directional derivative N-S (large scale)	$\sigma = 1.75\ km$
11	Directional derivative E-W (small scale)	$\sigma = 0.25\ km$
12	Directional derivative E-W (large scale)	$\sigma = 1.75\ km$

Table A2: Topographical predictors used as input features. A numerical ID has been assigned to each feature. Longitude, latitude and elevation have ID 0, 1 and 2, respectively. Adapted from [1]

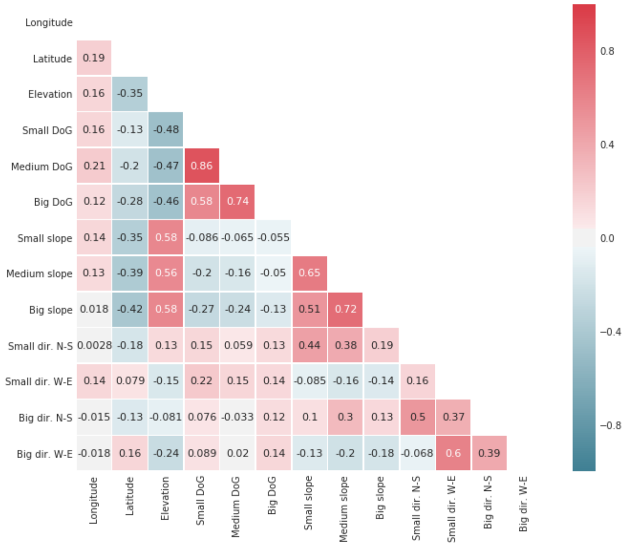

Fig. A5: **Correlation matrix .** Heatmap for the featurese included in the thirteen-dimensional input space.

A.3 Roughness estimation

Wind speed transformation in the Earth boundary layer under neutrally stable conditions and dynamic equilibrium has here been modelled using the generalized log-law presented in equation (23), which is based on the assumption that the mean velocity profile is a function of height, surface roughness, friction velocity, zero-plane displacement and von Karman's constant, usually assumed equal to 0.4 [2]. In this paper, roughness has been estimated based on the CLC by associating a roughness value to each of the land cover classes, as reported in Table A3. Such values have been widely used and validated, see e.g. [3].

Land Cover Class	h_0	Land Cover Class	h_0
Continuous urban fabric	1.2	Land principally occupied by agriculture	0.3
Discontinuous urban fabric	0.5	Broad-leaved forest	0.75
Industrial or commercial units	0.5	Coniferous forest	0.75
Roads and rail networks and associated land	0.075	Mixed forest	0.75
Ports areas	0.5	Natural grasslands	0.03
Airports	0.005	Moors and heatland	0.03
Mineral extraction sites	0.005	Transitional woodland-shrub	0.6
Construction sites	0.5	Beaches, dunes, sands	0.0003
Green urban areas	0.6	Bare rocks	0.005
Sport and leisure facilities	0.5	Sparsely vegetated areas	0.005
Non-irrigated arable land	0.05	Glaciers and perpetual snow	0.001
Vineyards	0.1	Inland marshes	0.05
Fruit trees and berry plantations	0.1	Water courses	0.00002
Pastures	0.03	Water bodies	0.00002
Complex cultivation patterns	0.3		

Table A3: **Roughness values.** The values are referred to the Corine Land Cover classes.

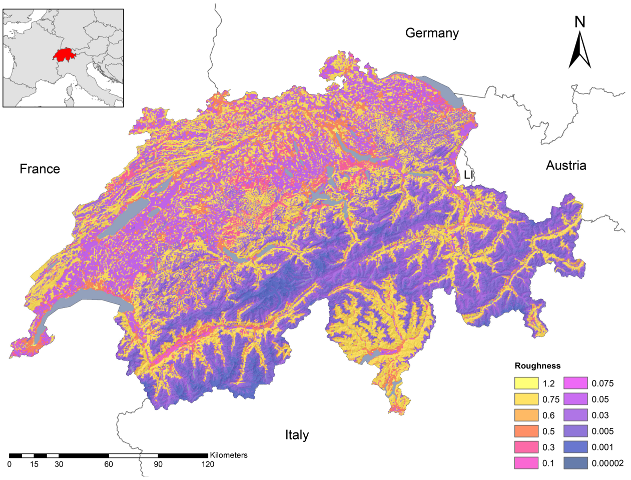

Fig. A6: **Roughness map.** The map is based on the Corine Land Cover 2018.

As a result, two roughness maps have been uses, one corresponding to the land covers mapped in the 2012 CLC, one for those mapped in the 2018 CLC data. The roughness map corresponding to the 2018 CLC is show in Figure A6.

Appendix B

B.1 ELM Tikhonov factor analysis

Regularised ELM can be intended as a ridge regression [4, 5] in a random feature space. This suggests that the Tikhonov factors can be used to increase the explainability of the spatio-temporal models. Tikhonov factors for the first 25 EOF components of each model are presented in Figure B7 as matrices with a linear colour scale depending on the magnitude order of α. In the Figure, a factor in yellow indicates the selection of a large regularisation parameter, which shrinks the output weights of ELM near to zero, hence suggesting the absence of structure in the corresponding modelled spatial coefficient map. It is worth noticing that the corresponding model variance will also tends to zero. Differently, dark tones in the Figure correspond to regularisation parameters closer to zero, hence to a behaviour closer to that of the classical LS procedure. This suggests that the regularised ELM is prone to consider a spatial structure for the corresponding EOF coefficients.

The matrix of the first model in Figure B7 tends to be less sparse with more recent data. This may be related to the increasing number of training stations over the different datasets. Reading vertically the matrices, hence looking at the columns, it is easy to verify how the first components provide the major contribution in terms of data variability and of spatially structured information. Reading now the matrices horizontally, hence looking at the rows, permits the detection of the variability within ensembles for some components of the first component group.

Other patterns can be identified for the single datasets. The MSWind 08-12 data in Figure B7a mainly takes advantage of the first five components, which from the EOF decomposition are known to cumulate 65% of data variability. Excepted for some isolated members in component 7 and 13, no other information is used by the model. Figure B7b shows how for the MSWind 13-16 data the first three components and the sixth one are chosen by the model for all members. Again, they represent about 65% of the variability. For the MSWind 17 data in Figure B7c components 1 to 6, 9, 11 and 17 are fully contributing to the model. They correspond to 73% of the variability. Components 8, 13, 19 20 and 24 are only partly contributing. All other components are automatically not considered due to the regularisation mechanism. For the three datasets, the very first component of the original data model (contains seasonal cycles which are weakly depending on space) is variable, hence denoting some fluctuations of the model. For the second model based on log-squared residuals, the difference between the regularization parameters are more neat among the components.

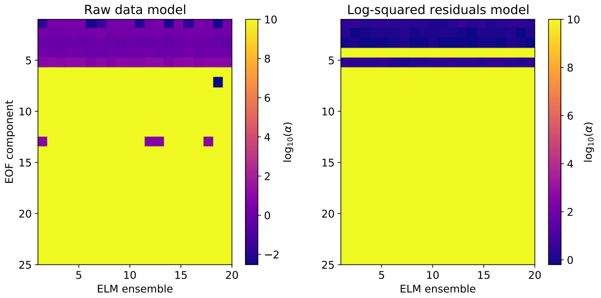

(a) MSWind 08-12.

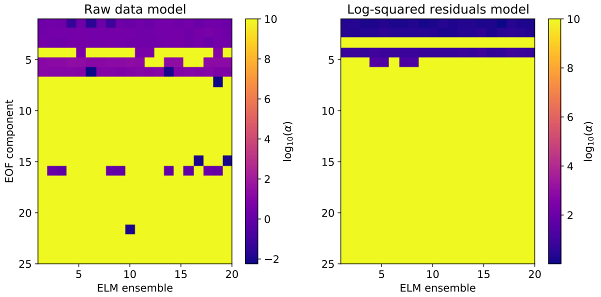

(b) MSWind 13-16.

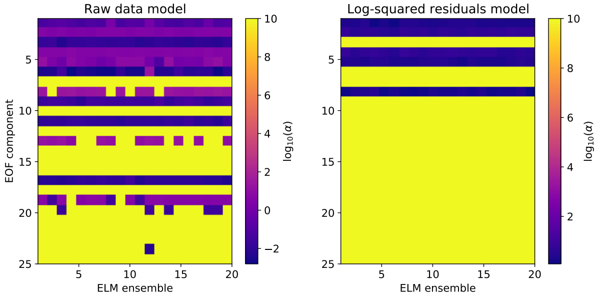

(c) MSWind 17.

Fig. B7: **Regularisation parameters for the first 25 components, for the 20 members of the ELM ensemble and for all models:** (left) The first spatio-temporal model is performed on the original wind speed data and provides the interpolated values and its model variance; (right) The second spatio-temporal model is performed on the log-squared residuals of the first one and provides the prediction variance.

B.2 Residuals Analysis

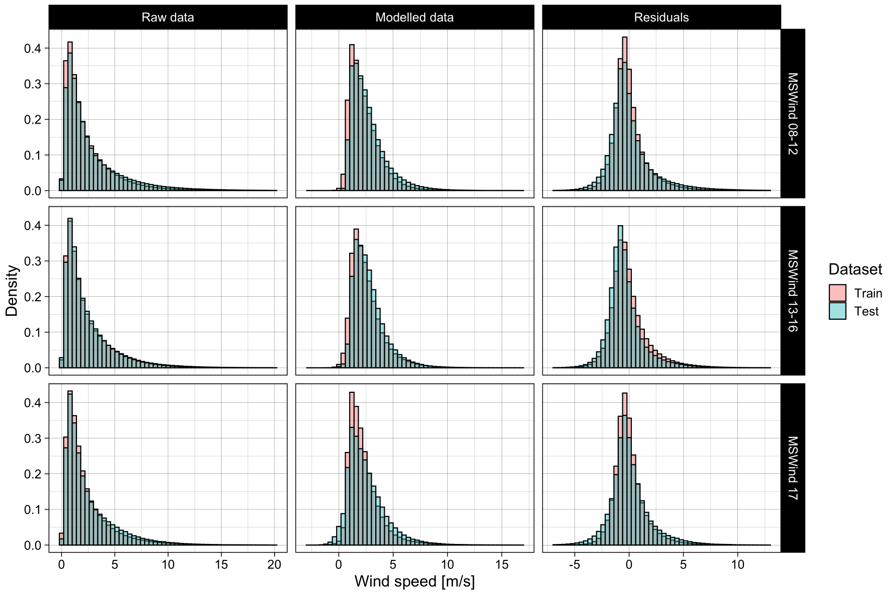

Fig. B8: **Histograms.** For visualisation purposes, the plots are zoomed in. The actual ranges and the means of each histogram are reported in Table B4

A careful analysis of the residuals is carried out. Figure B8 displays histograms of training and test set of the raw data, modelled data and residuals, for the three periods of study. Note that the plots are zoomed in for visualisation purposes and the actual ranges are reported in Table B4, together with the empirical means. The models predict negative values for the three datasets. Although a negative wind speed has no physical meaning, histograms show that it is happening quite rarely. These predicted values have been set to zero for the purpose of power estimation. The training residual means are all null and test ones are close to zero. However, each residual distribution has its mode below zero and is slightly skewed.

Spatio-temporal variography analysis is performed on the training data for some chosen months. Figure B9 visualise the semivariograms for raw data, model predictions and their residuals. The model reproduces well the spatio-temporal dependencies detected by variography for the selected months. The sill is lower for the modelled data, hence suggesting a substantial variability loss, possibly due to the noisy nature of the raw data. The semivariograms of the residuals are close to flat with a residual temporal structure. In the spatial axis, almost a pure nugget effect is observed, although a residual structure could subsist for January 2017. Globally, the patterns observed in the raw data semivariograms are reproduced in the corresponding semivariograms of

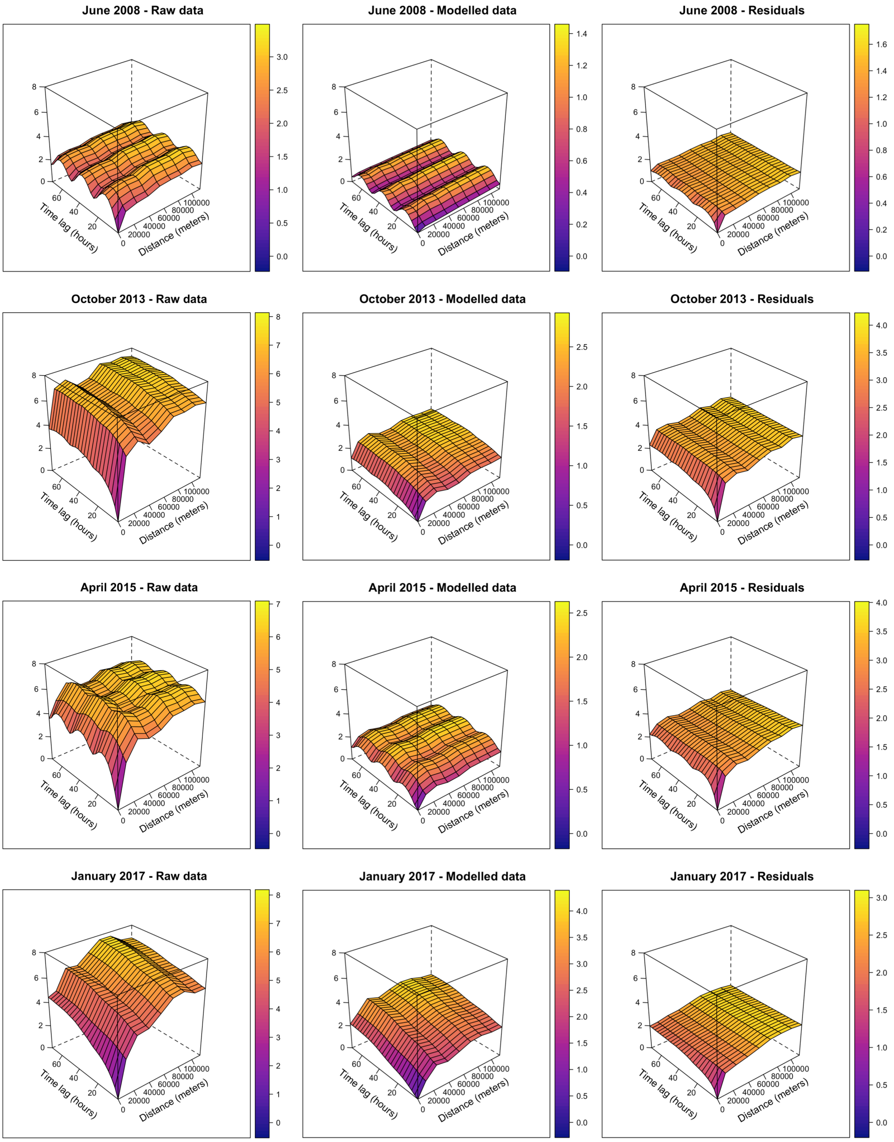

Fig. B9: **Variography for the spatio-temporal ELM modelling.** Spatio-temporal semivariograms have been computed on the training points for different months. The corresponding sample variance (or a priori variance, APV) can be found in Table B5.

modelled data, modulo the sill shift. This is even more striking by looking at longer temporal lags e.g. for January 2017, where a high similarity is observed between the semivariogram shapes, see Figure B10.

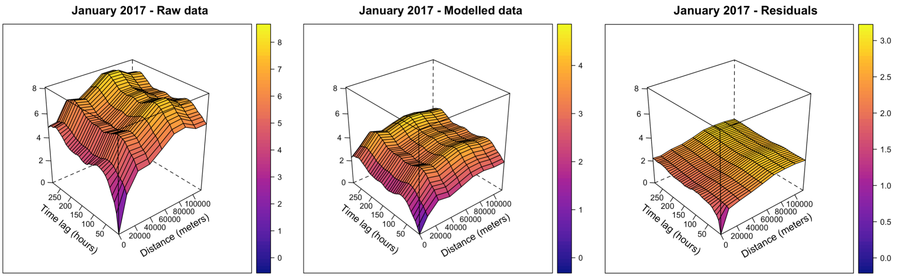

Fig. B10: **Temporally extended semivariogram for January 2017.** The same semivariogram of Figure B9 computed up to 12 days.

Spatial variography [6] is also performed on some spatial coefficients of EOF component before and after ELM modelling. Figure B11 displays such analysis for the MSWind 17 dataset for the first three components, which contain most of the variability. The (spatial) omnidirectional semivariogram is computed on the spatial coefficient obtained directly from the EOF decomposition of the original spatio-temporal data (in solid line). It shows the presence of the spatial structures in the first three components, although it is less pronounced for the very first component which is consistent with what was observed and discussed so far in Figure B7. Figure B11 also shows the semivariograms of the residuals obtained from the spatial modelling with ELM ensembles (in dashed line), which are close to pure nugget effects. This has two consequences. First, the spatial modelling component by component seems to correctly extract the spatial structures from them. Second, this suggests that the heteroskedastic variance estimate of the ELM ensembles satisfies its independence assumption — actually, it is sufficient to assume a vanishing covariance for this estimation, see [7] — and hence is appropriate. Finally, the cross-variograms between the residuals of component pairs fluctuate near zero (in dashed-dotted line). Comparing to the corresponding semivariograms, this indicates that the correlation between component residual pairs is very weak [8] and then satisfy the additional assumption for spatio-temporal model variance estimation which is necessary to ensure that no variability comes from spatial model interactions. The other components, which contain far less variability, can be exempted from such analysis without too many risks.

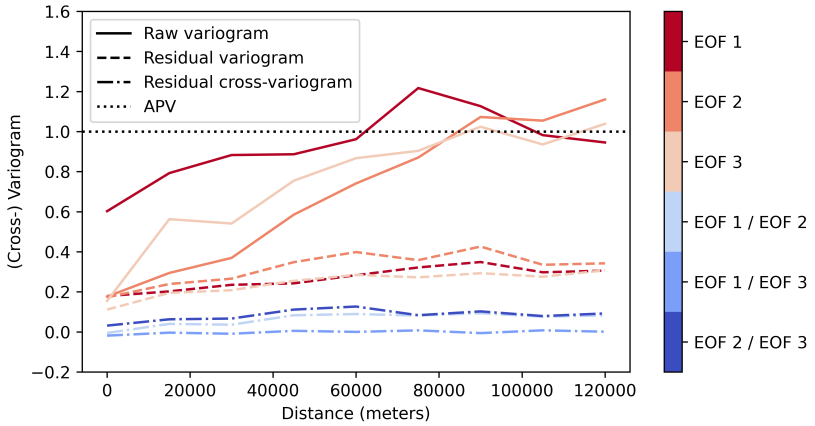

Fig. B11: **Spatial variography of the first three components for MSWind 17.** Spatial semivariograms of the raw data (solid line) and residuals (dashed line) after spatial modelling with ELM. Cross-semivariograms between residuals from two different components (dashed-dotted line). All components have a unitary sample variance (or a priori variance, APV) (dotted line) due to the normalisation of the spatial coefficients during the EOF decomposition procedure.

		**Training set**			**Test set**		
**Dataset**	**Statistic**	Raw	Mod.	Res.	Raw	Mod.	Res.
MSWind 08-12	Min.	0.10	-0.97	-9.08	0.10	-0.25	-11.83
	Mean	2.33	2.33	0.00	2.81	2.71	0.10
	Max.	38.08	20.22	22.13	42.85	18.30	34.54
MSWind 13-16	Min.	0.10	-1.26	-11.62	0.10	-0.71	-10.11
	Mean	2.56	2.56	0.00	2.42	2.78	-0.37
	Max.	40.43	19.59	29.22	25.13	16.88	16.89
MSWind 17	Min.	0.10	-2.88	-8.66	0.10	-2.69	-17.18
	Mean	2.30	2.30	0.00	2.56	2.50	0.06
	Max.	34.15	26.32	24.44	34.67	20.35	25.04

Table B4: **Summary statistics.** The minimum, the empirical mean, and the maximum are computed on the original data before modelling (Raw), the modelled data (Mod.) and residuals (Res.) for the training and test stations of the three datasets.

		Raw data	Modelled data	Residuals
June	2008	2.85	1.01	1.51
October	2013	6.66	2.34	3.46
April	2015	6.62	2.56	3.46
January	2017	6.61	3.40	2.71

Table B5: **APVs of the spatio-temporal semivariograms of Figure B9.** The sample variance (or a priori variance, APV) is computed on the training data.

Appendix C

C.1 Definition of restriction zones

As explained in Section 3.3, the restriction zones shown in Table 3 are based on the framework for wind energy planning in Switzerland developed by the Swiss Federal Office of Spatial Development (ARE) [9]. It divides, on a scale of 500×500 m^2, between buffered building zones, protected areas, areas to be excluded in principle (considered *prohibited*), areas with a potential balancing of interests in case of national interest (considered *restricted*), areas subject to inter-authority coordination and other areas (no restriction assumed).

Forests are considered here as a separate category, as wind turbine installation may be possible (unless other restrictions apply), but these zones are considered to be more vulnerable than other areas. Furthermore, all areas at altitudes above 3,000 meters are considered *prohibited* (based on a digital terrain model [10]), as they mark highly alpine terrain that is typically difficult to access. Areas above 2,500 meters of altitude are categorised as *restricted*, as currently no installations are found at higher altitudes and wind turbines in these regions may be difficult to install and maintain.

References

[1] Robert, S., Foresti, L., Kanevski, M.: Spatial prediction of monthly wind speeds in complex terrain with adaptive general regression neural networks. International Journal of Climatology **33**(7), 1793–1804 (2013) https://rmets.onlinelibrary.wiley.com/doi/pdf/10.1002/joc.3550. https://doi.org/10.1002/joc.3550

[2] Grassi, S., Veronesi, F., Raubal, M.: Satellite remote sensed data to improve the accuracy of statistical models for wind resource assessment. In: European Wind Energy Association Annual Conference and Exhibition 2015 (2015)

[3] Brown, O.W., Hugenholtz, C.H.: Estimating aerodynamic roughness (zo) in mixed grassland prairie with airborne lidar. Canadian Journal of Remote Sensing **37**(4), 422–428 (2011)

[4] James, G., Witten, D., Hastie, T., Tibshirani, R.: An introduction to statistical learning **112** (2013)

[5] Hastie, T., Tibshirani, R., Friedman, J.H.: The elements of statistical learning: Data mining, inference, and prediction (2009)

[6] Kanevski, M., Maignan, M.: Analysis and modelling of spatial environmental data **6501** (2004)

[7] Guignard, F., Amato, F., Kanevski, M.: Uncertainty quantification in extreme learning machine: Analytical developments, variance estimates and confidence intervals. Neurocomputing **456**, 436–449 (2021). https://doi.org/10.1016/j.neucom.2021.04.027

[8] Chiles, J.-P., Delfiner, P.: Geostatistics: modeling spatial uncertainty **497** (2009)

[9] für Raumentwicklung ARE, B.: Konzept Windenergie. Basis zur Berücksichtigung der Bundesinteressen bei der Planung von Windenergieanlagen. Technical report, Bern, Switzerland (2020)

[10] Swisstopo: swissALTI3D - The high precision digital elevation model of Switzerland (2017). https://shop.swisstopo.admin.ch/en/products/height_models/alti3D Accessed 2019-08-13
